# Upregulation of the Long Non-coding RNA LINC01480 Is Associated With Immune Infiltration in Coronary Artery Disease Based on an Immune-Related lncRNA-mRNA Co-expression Network

**DOI:** 10.3389/fcvm.2022.724262

**Published:** 2022-04-26

**Authors:** Ting Xiong, Botao Xiao, Yueheng Wu, Yunfeng Liu, Quhuan Li

**Affiliations:** ^1^School of Bioscience and Bioengineering, South China University of Technology, Guangzhou, China; ^2^Guangdong Provincial Engineering and Technology Research Center of Biopharmaceuticals, South China University of Technology, Guangzhou, China; ^3^Guangdong Cardiovascular Institute, Guangdong Provincial Key Laboratory of South China Structural Heart Disease, Guangdong General Hospital, Guangzhou, China

**Keywords:** coronary artery disease, atherosclerosis, ischemic cardiomyopathy, long noncoding RNA, immune molecule

## Abstract

Coronary artery disease (CAD) is considered one of the leading causes of death worldwide. Although dysregulation of long non-coding RNAs (lncRNAs) has been reported to be associated with the initiation and progression of CAD, the knowledge regarding their specific functions as well their physiological/pathological significance in CAD is very limited. In this study, we aimed to systematically analyze immune-related lncRNAs in CAD and explore the relationship between key immune-related lncRNAs and the immune cell infiltration process. Based on differential expression analysis of mRNAs and lncRNAs, an immune-related lncRNA-mRNA weighted gene co-expression network containing 377 lncRNAs and 119 mRNAs was constructed. LINC01480 and AL359237.1 were identified as the hub immune-related lncRNAs in CAD using the random forest-recursive feature elimination and least absolute shrinkage and selection operator logistic regression. Furthermore, 93 CAD samples were divided into two subgroups according to the expression values of LINC01480 and AL359237.1 by consensus clustering analysis. By performing gene set enrichment analysis, we found that cluster 2 enriched more cardiovascular risk pathways than cluster 1. The immune cell infiltration analysis of ischemic cardiomyopathy (ICM; an advanced stage of CAD) samples revealed that the proportion of macrophage M2 was upregulated in the LINC01480 highly expressed samples, thus suggesting that LINC01480 plays a protective role in the progression of ICM. Based on the findings of this study, lncRNA LINC01480 may be used as a novel biomarker and therapeutic target for CAD.

## Introduction

Coronary artery disease (CAD) is one of the most prevalent cardiovascular diseases (CVDs) and one of the leading causes of death worldwide (1). At present, an increasing number of young people have CAD. According to the clinical symptoms, the World Health Organization classified CADs into five types in 1979 (2), namely latent CAD, stable or unstable angina, myocardial infarction, ischemic cardiomyopathy (ICM), and sudden cardiac death (3). Among them, ICM is considered a special type or an advanced stage of CAD (4, 5). The development of CAD is regulated by a combination of genetic and lifestyle-related factors (6), such as environmental and social stress, smoking, unhealthy diet, and chemical contaminant exposure.

The underlying pathological process of CAD involves atherosclerosis, which is considered a chronic inflammatory disease (7). Accumulation of large amounts of low-density lipoprotein (LDL) in the endarterium initiates the formation of atherosclerotic plaques. Moreover, infiltrating LDL particles may be oxidatively modified in the subendothelial space, further aggravating endothelial cell dysfunction. Monocytes/lymphocytes are then recruited to initiate the process of transendothelial migration to the lesion site *via* adhesion molecules expressed by endothelial cells. Infiltrating monocytes differentiate into macrophages and subsequently internalize the abundant oxidized LDL (OxLDL), finally differentiating into foam cells. The cholesterol-rich foam cell finally dies and becomes an essential component of the plaque necrotic core. Aberrant proliferation and phenotypic transformation of smooth muscle cells and collagen synthesis are important factors in plaque stability (8), which is associated with fibrous cap development. The characteristics of plaques prone to thrombotic events involve a large lipid-filled necrotic core, thin fibrous cap, and persistent inflammatory responses. The clinical symptoms of atherosclerosis include stroke or myocardial infarction, in which thrombotic events occur due to plaque rupture or endothelial erosion. Thus, dysregulation of the immune system is a major cause of atherosclerosis development. Moreover, targeting of specific immune molecules involved in atherosclerosis revealed that they help regulate immune system homeostasis and, thus, disease progression (9).

Long non-coding RNAs (lncRNAs) do not encode proteins. They have a length of > 200 nucleotides and are stable in the peripheral blood or other body fluids. Based on their genomic location, lncRNAs can be divided into six major classes: sense, sense intronic, antisense, bidirectional, enhancer, or intergenic lncRNAs (10, 11). Previously, they were considered “evolutionary junk.” However, increasing evidence has shown that lncRNAs play important roles in multiple diseases, including heart diseases, cancers, and diabetic nephropathy, *via* epigenetic modifications and transcriptional or post-transcriptional regulation mechanisms (12, 13).

Accumulating evidence has shown that some differentially expressed lncRNAs are associated with atherosclerosis initiation and progression (14). The antisense non-coding RNA in the INK4 locus (ANRIL) is located at the cardiovascular disease risk site on chromosome 9p21 (15). ANRIL knockdown and overexpression experiments confirmed that it plays a role in the regulation of the activities of endothelial cells by modulating the expression levels of *CLIP1*, *EZR*, and *LYVE1* (16). Furthermore, it serves as a modular scaffold of the WDR5-HDAC3 complex, which promotes the phenotypic transition of vascular smooth muscle cells (17). Metastasis-associated lung adenocarcinoma transcript 1 (MALAT1) is upregulated in CAD blood samples and affects disease development *via* the mammalian target of rapamycin (mTOR) signaling pathway (18). Furthermore, MALAT1 may facilitate OxLDL-induced endothelial inflammation by upregulating the expression of the miR-181b target gene *TOX* (19). Expression level of myocardial infarction associated transcript (MIAT) is elevated in the peripheral blood of CAD patients (20); it could induce *CD47* expression and thereby inhibit efferocytosis in advanced atherosclerosis by acting as an miR-149-5p sponge (21). Moreover, studies on atherosclerosis mice suggested that MIAT could activate the PI3K/Akt signaling pathway to aggravate atherosclerosis damage (22). These findings suggest that lncRNAs positively participate in modulating atherosclerotic plaques and thus play a role in CAD development.

To date, although some lncRNAs have been reported to be associated with atherosclerosis, there has been no integrated analysis of the immune-related lncRNAs in CAD. In this study, we constructed an intricate immune-related biological network that was associated with the progression of CAD. Furthermore, we identified two novel immune-related lncRNAs as hub molecules and found that they were closely related to the heterogeneity observed in CAD. Interestingly, the expression of LINC01480 was upregulated in the ICM tissue samples, indicating that it may be a potential target to regulate the immune cell infiltration. Overall, the findings of our study greatly improve our understanding of the lncRNA-related immune mechanisms in CAD. In addition, the novel lncRNA LINC01480 identified in this study will help to further understand the immune cell infiltration process in ICM and provide new insights for the development of effective drugs for the treatment of ICM.

## Materials and Methods

### Data Source

Five datasets were used in this study. The CAD-related dataset derived from the peripheral blood mononuclear cells (PBMCs; GSE113079) and the ICM-related datasets derived from the left ventricles (GSE48166, GSE116250, GSE46224, and GSE120825) were downloaded from the Gene Expression Omnibus (GEO) database^1^ (23). The GSE113079 included 93 CADs and 48 healthy controls (24). The GSE48166 included 15 ICMs and 15 healthy controls. The GSE116250 contained 13 ICMs and 14 healthy controls (25). The GSE46224 included eight ICMs and eight healthy controls (26). In addition, the GSE120825 included five ICMs and five healthy controls (27). The basic information of these datasets is provided in Supplementary Table 1. Moreover, signature sets of the immune-related genes (IRGs) were obtained from the ImmPort database^2^ (28). The reference human genome (GRCh38) and the genome annotation file (Humo_sapiens.GRCh38.99.gtf) were downloaded from the Ensembl database.

### Data Preprocessing

The GSE113079 platform was GPL20115 (Agilent-067406 Human CBC lncRNA + mRNA microarray V4.0) and it was reannotated to obtain unique lncRNA and mRNA probe sets. First, the probe sequences were aligned to the reference human genome (GRCh38) without a mismatch. Then, the Ensembl IDs were transformed to gene symbols using a human genome annotation file (Humo_sapiens.GRCh38.99.gtf) and only those annotated as “lncRNA” or “protein_coding” were retained. If one probe corresponded to multiple Ensembl IDs, it was deleted. Finally, the reannotated GPL20115 platform contained 25530 mRNA probes and 26180 lncRNA probes. The GSE113079 expression profile was annotated using the reannotated GPL20115 platform.

Raw RNA sequencing data (GSE48166, GSE116250, GSE46224, and GSE120825) were transformed into counts and merged. Here, FastQC was used to evaluate the quality of the raw reads. Adaptors and low-quality bases were filtered using Trimmomatic software. Then, the clean reads were aligned to the reference genome *via* HISAT2. Sequence alignment/map (.SAM) format files were converted into binary alignments/maps (.BAM) format using SAMtools. Eventually, count data were further obtained using htseq-count. The principle that Ensembl IDs were transformed to gene symbols was the same as in GSE113079. In addition, the RNAseq datasets were combined and the batch effects were corrected for subsequent analyses. First, the counts from different datasets were normalized individually *via* variance stabilizing transformed (VST) function in the DESeq2 package. The removeBatchEffect function of the limma package was used to correct the batch effect (29), and the removal of the batch effects was visualized *via* principal component analysis (PCA), which was performed *via* the PCA function in the factoextra package.

### Identification of the Differentially Expressed Long Non-coding RNAs and mRNAs

The GSE113079 gene expression matrix was normalized using the normalizeBetweenArrays function of the limma package and annotated using the reannotated GPL20115 platform. If multiple probes corresponded to the same gene, the probe with the maximum average expression was reserved. Differences in the expression levels of the lncRNAs and mRNAs between the CAD and healthy samples were analyzed using the limma package. | log_2_ (FC)| > 0.8, and adj.P.Value < 0.001 were used to select the DELs. *P* < 0.05 was the cut-off threshold for differentially expressed mRNAs (DEMs). Differentially expressed immune-related genes (DEIRGs) were identified from the overlapping regions of DEMs and IRGs. Furthermore, the Metascape database^3^ was used to analyze the biological function of DEIRGs. The statistical significance was set at *P* < 0.01.

### Immune-Related Long Non-coding RNA-mRNA Co-expression Network in Coronary Artery Disease

We used weighted gene co-expression network analysis (WGCNA) to construct a lncRNA-mRNA co-expression network to study the relationship between the DELs and DEIRGs (30). We selected β = 3 as the soft threshold. A weighted correlation coefficient > 0.4 was the criterion for the co-expression network, which was visualized using Cytoscape_v3.7.2 (31).

### Screening Hub Immune-Related Long Non-coding RNA*s*

The random forest-recursive feature elimination (RF-RFE) algorithms with 10-fold cross validation or Bootstrap, and the least absolute shrinkage and selection operator (LASSO) logistic regression with 10-fold cross validation were used for feature selection of the hub immune-related lncRNA molecules for CAD (32, 33), and they were achieved *via* caret and glmnet packages, respectively. Eventually, the overlapping molecules screened by both algorithms were regarded as crucial molecules for subsequent analysis.

### Consensus Clustering Analysis

A consensus clustering algorithm was applied to explore the heterogeneity according to the expression values of the hub immune-related lncRNA molecules. Clustering was performed using unsupervised clustering methods (K-means) with the Euclidean distance, and this procedure was completed using the ConsensuClusterPlus R package. The maximum cluster number was set to 10 and the final cluster number was determined using the consensus matrix. In addition, PCA and Hierachical clustering (Hclust) were used to verify the rationality of clustering. Hclust was performed by using the Hclust function with the Euclidean distance based on the stats R packages.

### Gene Set Enrichment Analysis and Gene Set Variation Analysis

GSEA was performed to investigate the specific pathways of subgroups by comparing the CAD samples in each subgroup with the normal controls (34). To preliminary speculate the underling pathways, GSVA was firstly used to assess the relative gene set scores for the samples of ICM datasets. Subsequently, Spearman’s correlation was calculated to explore the relationship between expression value of LINC01480 and the GSVA score of pathways. GSEA was performed using GSEA v4.0.3,^4^ and a value of *P* < 0.05, was used to screen the significant differential pathways after performing 1000 permutations. The GSVA scores of the ICM datasets were calculated using the gsva function in the GSVA package. In addition, the Kyoto Encyclopedia of Genes and Genomes (KEGG) gene set collection (c2.cp.kegg.v7.1.symbols.gmt) was used as the input file for both GSEA and GSVA analyses.

Furthermore, we also predicted the function of LINC01480 by analyzing its co-expression genes. These co-expression genes were determined by calculating the Pearson’s correlation coefficient of expression values between LINC01480 and mRNAs in ICMs samples. Pearson’s correlation coefficient > 0.3 and *P* < 0.05 were used to select the positive co-expression genes of LINC01480, and negative co-expression genes of LINC01480 were screened by Pearson’s correlation coefficient < –0.3 and *P* < 0.05. Finally, the KEGG pathway analysis was performed using the co-expression genes on the online website DAVID (DAVID Functional Annotation Bioinformatics Microarray Analysis (ncifcrf.gov)), and a value of *P* < 0.01 was considered statistically significant.

### Evaluation of Immune Cell Infiltration by CIBERSORTx Analysis

We uploaded the normalized gene expression values of ICM datasets onto the online website of CIBERSORTx (35), and only the samples with *P* < 0.05 were reserved for further analysis. Subsequently, the proportion of 22 types of infiltrating immune cells was calculated according to the LM22 file containing the expression values of 547 genes. The close relationship between lncRNAs and proportion of immune cells was evaluated using Spearman’s correlation coefficient, and a value of *P* < 0.05 was considered statistically significant.

### Statistical Analysis

R software was used to evaluate the correlation between the lncRNAs and GSVA scores using Spearman’s correlation analysis. The statistical significance was set at *P* < 0.01.

### Overall Analysis Flowchart

**FIGURE 1:**
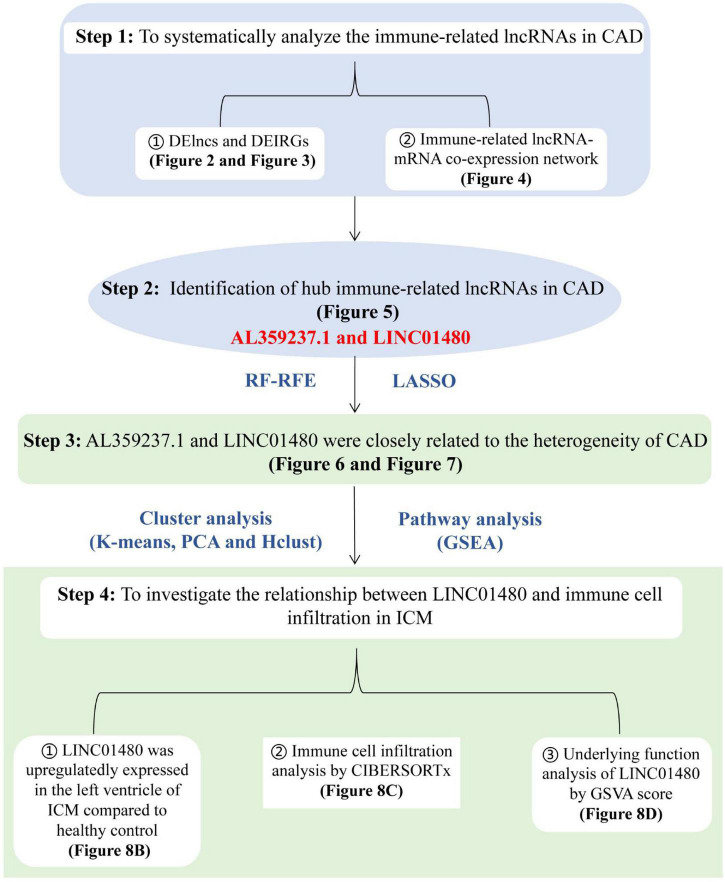
Flowchart of the data analysis. The analytical procedure for step1 to step3 was performed based on the microarray dataset [GSE113079, derived from PBMCs of coronary artery disease (CADs) and healthy controls], and the analysis of step4 was based on the RNAseq datasets (GSE48166, GSE116250, GSE46224, GSE120825, derived from left ventricles of ICMs and healthy controls). CAD, coronary artery disease; ICM, ischemic cardiomyopathy; DELncs, differentially expressed long non-coding RNAs; DEIRGs, differentially expressed immune-related genes; lncRNA, long non-coding RNA; mRNA, messenger RNA; RF-RFE, random forest-recursive feature elimination; LASSO, the least absolute shrinkage and selection operator; PCA, principal component analysis; Hclust, hierarchical clustering; GSEA, gene set enrichment analysis; GSVA, gene set variable analysis; RNAseq, RNA sequencing.

To systematically analyze immune-related lncRNAs and identify hub immune-related lncRNAs in CAD, we used a systematic bioinformatics method to analyze the CAD GSE113079 dataset. The overall flowchart of this study is shown in Figure 1. First, we identified DELs and DEIRGs between the CAD and healthy samples. Subsequently, lncRNA expression patterns were explored by constructing an immune-related lncRNA-mRNA weighted co-expression network. The potential biological functions of DEIRGs were investigated *via* the Metascape online website. The RF-RFE and LASSO logistic regression algorithms were then combined to screen for crucial immune-related lncRNAs. Furthermore, a consensus clustering analysis was used to study the heterogeneity of CAD disease; GSEA was performed to investigate the biological differences between subgroups and controls. Finally, CIBERSORTx was utilized to explore the relationship between hub lncRNAs and the proportion of infiltrating immune cells in ICM samples, and GSVA was used to study the biological pathways involved in ICM.

## Results

### DEL and DEM Analysis and Differentially Expressed Immune-Related Gene Identification

**FIGURE 2:**
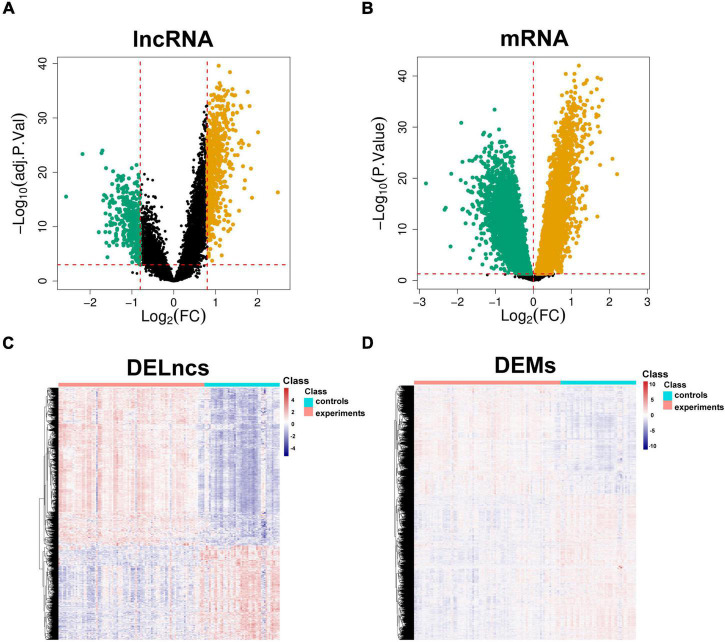
Identification of aberrantly expressed molecules. Volcano plots of DELncs **(A)** and DEMs **(B)** in the CAD samples and controls. Heatmaps of DELncs **(C)** and DEMs **(D)**. Brown and green points correspond to upregulated and downregulated molecules, respectively, and black points indicate that there are no differences in the expression levels of the molecules. DELncs, differentially expressed lncRNAs; DEMs, differentially expressed mRNAs; CAD, coronary artery disease.

**FIGURE 3:**
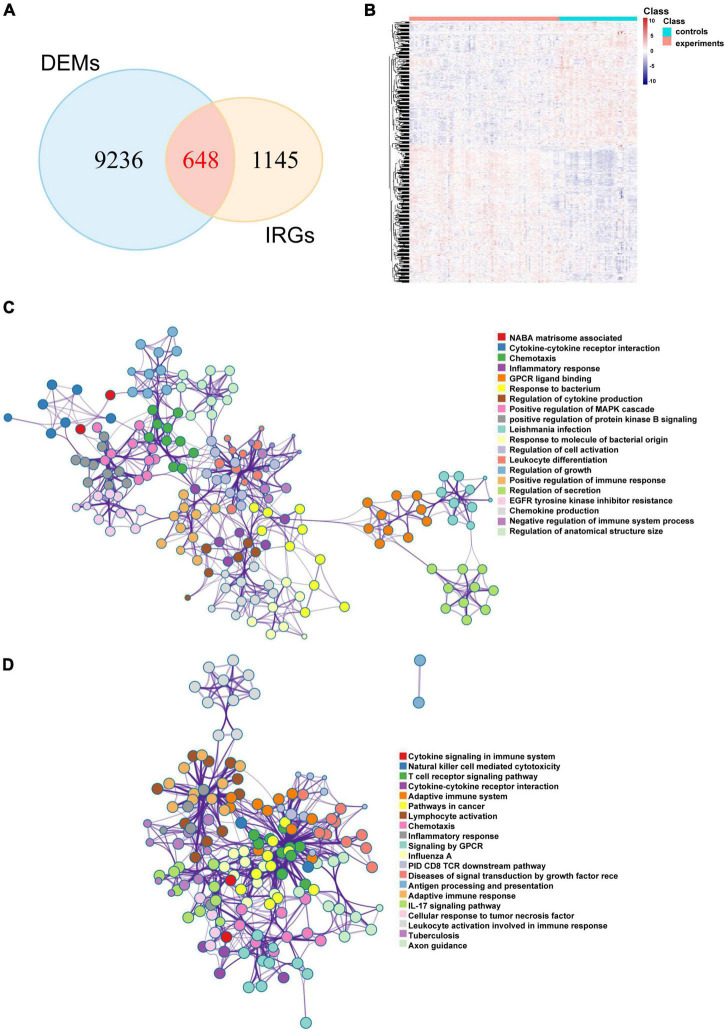
Potential biological pathways of DEIRGs in CAD. **(A)** Venn diagram of the intersections between the DEMs and IRGs and the heatmap of DEIRGs. **(B)** The network of enriched terms of upregulated DEIRGs **(C)** and downregulated DEIRGs **(D)** *via* the Metascape online website; nodes of the same color belong to the same term. DEIRGs: differentially expressed immune-related genes; CAD, coronary artery disease; DEMs, differentially expressed mRNAs; IRGs, immune-related genes.

The limma package was used to analyze the microarray data to identify the significantly expressed molecules in the CAD samples. Subsequently, volcano plots and heatmaps were constructed to visualize the dysregulated lncRNAs (Figures 2A,C) and mRNAs (Figures 2B,D). In total, 978 DELs (612 upregulated and 366 downregulated) and 9884 DEMs (4252 upregulated and 5632 downregulated) were identified, and the top 20 upregulated and downregulated mRNAs and lncRNAs are described in Supplementary Tables 2 and 3. Moreover, 648 DEIRGs were extracted from the intersection of the DEMs and IRGs (Figures 3A,B).

### Functional Enrichment Analysis of Differentially Expressed Immune-Related Genes

Metascape is a user-friendly and comprehensive database that reveals protein interactions and underlying biological mechanisms. To analyze the underlying roles of the DEIRGs in CAD, we used the Metascape online website to perform Gene Ontology (GO) term and KEGG pathway analyses. The results suggested that both upregulated and downregulated immune mRNAs were enriched in immune-related pathways. For example, upregulated immune mRNAs were involved in cytokine-cytokine receptor interaction, inflammatory response, leukocyte differentiation, and positive regulation of the immune response (Figure 3C). Downregulated immune mRNAs were related to cytokine signaling in the cytokine signaling immune system, natural killer (NK) cell mediated cytotoxicity, T cell receptor signaling pathway, and so on (Figure 3D). Consistent with previous reports, the results indicated that immune-related genes and pathways are positively involved in CAD development.

### Construction of the Immune-Related Long Non-coding RNA-mRNA Weighted Co-expression Network

To explore the role of immune-related lncRNAs in CAD, we constructed an lncRNA-mRNA co-expression network between DELs and DEIRGs using WGCNA. WGCNA is frequently used to investigate the complicated relationships among genes and uses a soft threshold to construct a scale-free topology network to emphasize essential molecules.

**FIGURE 4:**
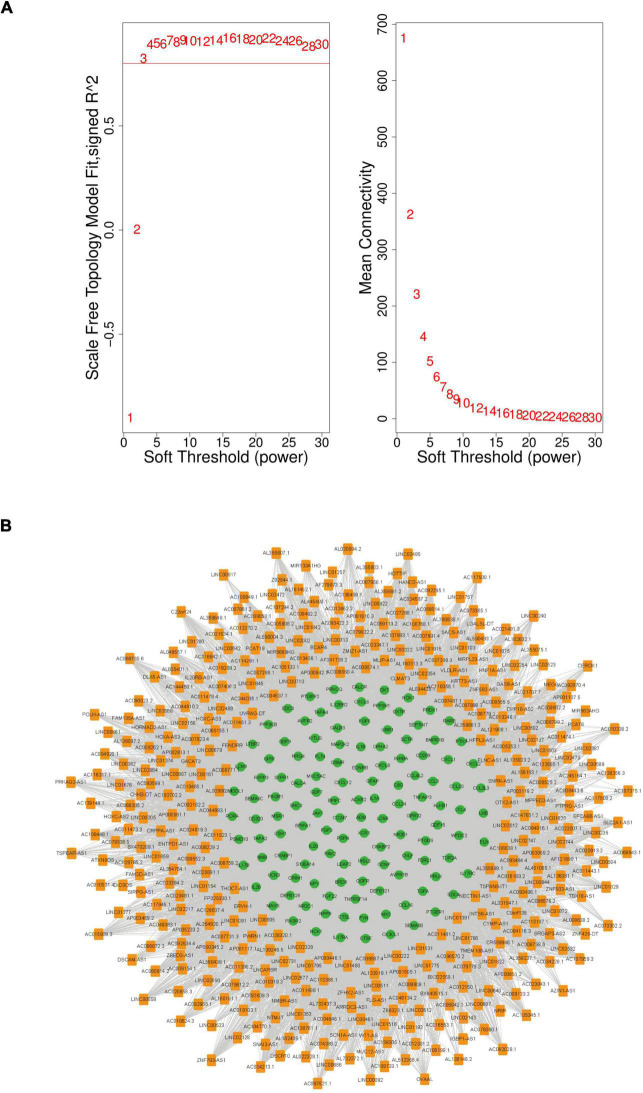
The immune-related lncRNA-mRNA weighted gene co-expression network in CAD. **(A)** Selection of the soft threshold to analyze the network topology; if a soft-threshold power of 3 is selected, the appropriate scale-free fit index can be attained. **(B)** The immune-related lncRNA-mRNA weighted co-expression network including 496 network nodes (377 lncRNAs and 119 mRNAs). Orange nodes that denote lncRNAs and green nodes that denote mRNAs. CAD, coronary artery disease; lncRNA, long non-coding RNA.

First, the power value was determined. A power value equal to 3 led to a scale-free topology model fit index > 0.8 and a high average connectivity degree. Therefore, this power value was used for the weighted correlation analysis (Figure 4A). In this study, lncRNAs with a high degree of ≥ 10 were selected to construct the immune-related co-expression network, which comprised 377 lncRNAs and 119 mRNAs (Figure 4B). Furthermore, lncRNAs from the immune-related co-expression network were regarded as immune-related molecules in CAD.

### Screening the Hub Immune-Related Long Non-coding RNA Molecules in Coronary Artery Disease

**FIGURE 5:**
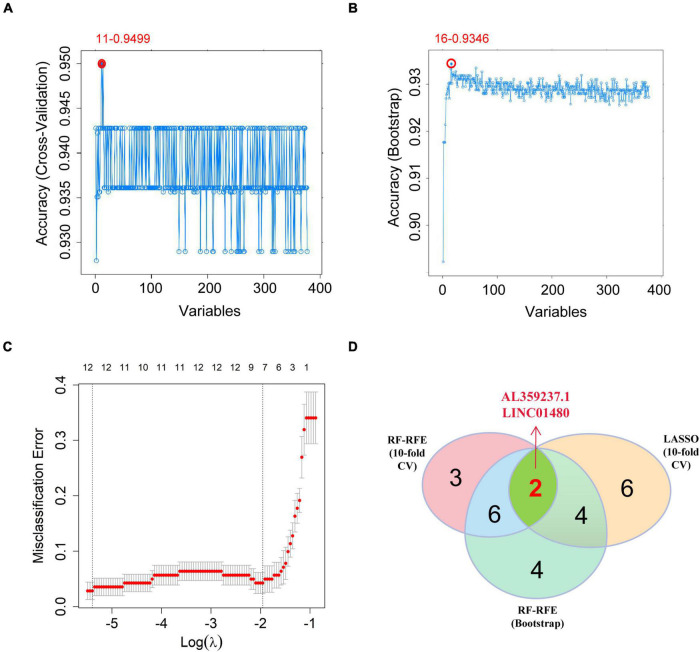
Screening the hub immune-related lncRNAs. **(A)** RF-RFE algorithm to screen the immune-related lncRNAs; at the highest accuracy level, the minimum number of variables was 11. **(B)** The minimum number of variables was 16 based on Bootstrap. **(C)** 10-fold cross-validation for the penalty parameters-lambda selection in the LASSO logistic regression model; 12 variables were reserved when lambda.min was selected. **(D)** Venn diagram showing the intersections between the hub immune-related lncRNAs obtained by the three algorithms. LASSO, the least absolute shrinkage and selection operator; lncRNAs, long non-coding RNAs; RF-RFE, random forest-recursive feature elimination.

To screen out the lncRNAs that are crucial for the occurrence and development of CAD, we combined RF-RFE and LASSO logistic regression to perform feature selection. Compared with cross-validation, Bootstrap can avoid the problem of reducing the number of samples by putting them back. Moreover, Bootstrap can also increase the randomness of data. Here, we used the RF-RFE algorithm with 10-fold cross validation to identify 11 immune-related lncRNAs as candidate hub molecules (Figure 5A), while 16 immune-related lncRNAs were identified using the RF-RFE algorithm with Bootstrap (Figure 5B). Furthermore, 12 immune-related lncRNAs were uncovered through LASSO logistic regression algorithm with 10-fold cross validation (Figure 5C). By overlapping the molecules selected by the three algorithms, we obtained two lncRNAs (LINC01480 and AL359237.1), which were regarded as the hub lncRNAs for subsequent analysis (Figure 5D and Supplementary Table 4).

### Consensus Clustering Analysis and Gene Set Enrichment Analysis

In clinical research, it has been found that there is heterogeneity in CAD. Therefore, it is of great significance to classify CAD samples for subsequent subgroup targeted therapy.

**FIGURE 6:**
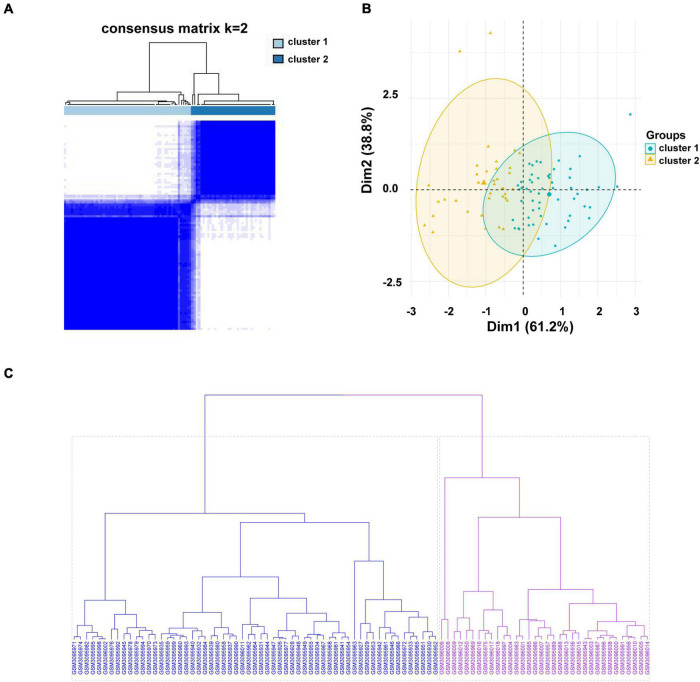
Consensus clustering analysis of the hub immune-related lncRNAs in the CAD samples. **(A)** The heatmap of the consensus matrix with a cluster count of 2. **(B)** Two-dimensional PCA cluster plot of the CAD samples; green points denote the samples of cluster 1 and yellow triangles denote to the samples of cluster 2. **(C)** Unsupervised hierarchical clustering. Clustering was performed for 93 CAD samples based on the expression values of LINC01480 and AL359237.1. The cluster 1 containing 59 samples is colored in blue, and cluster 2 including 34 samples is colored in purple. lncRNA, long non-coding RNA; CAD, coronary artery disease; PCA, principal component analysis; Hclust, hierarchical clustering.

The gene expression profiles of LINC01480 and AL359237.1, were used to classify 93 CAD samples into subgroups using the unsupervised clustering method (consensus clustering analysis). The consensus matrix indicated that k = 2 was more appropriate, in which cluster 1 contained 56 samples and cluster 2 contained 37 samples (Figure 6A). PCA cluster analysis can be applied to compare the similarities and differences between biological samples. The PCA diagram also showed that there was a significant difference between clusters 1 and 2 (Figure 6B). Furthermore, the result of Hierarchical clustering (Hclust) validated reasonability of classification that the CAD samples were divided into two groups above (Figure 6C), in which cluster 1 contained 59 CAD samples, and cluster 2 included 34 CAD samples. The similarity of classification between K-means and Hclust methods reached 96.7%, which suggested that our results are credible (Supplementary Table 5).

**FIGURE 7:**
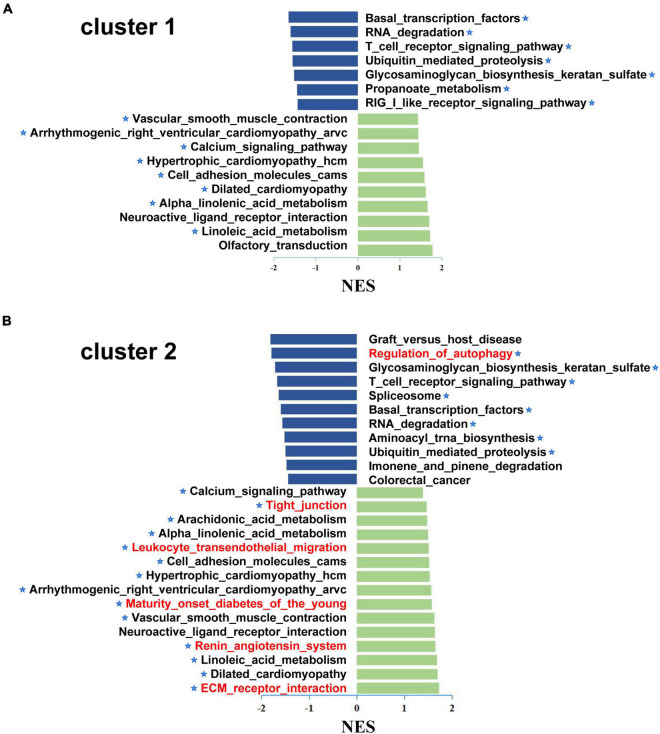
The Gene set enrichment analysis (GSEA) of different subgroup in the CAD samples. GSEA was used to explore the differences between the biological pathways of cluster 1 **(A)** and cluster 2 **(B)**; x-axis represents the NES and the asterisks denote the pathways associated with cardiovascular disease. In addition, the unique aberrant pathways were marked in red in cluster 2 compared to cluster 1. GSEA, gene set enrichment analysis; NES, normalized enrichment score.

To further analyze the differences between subgroups, GSEA was performed to investigate the underlying biological pathways. The results suggested that several pathways were activated in both subgroups, including vascular smooth muscle contraction, arrhythmogenic right ventricular cardiomyopathy, calcium signaling pathway, hypertrophic cardiomyopathy, etc. Some pathways were inhibited, including glycosaminoglycan biosynthesis, keratan sulfate biosynthesis, T cell receptor signaling pathway, and basal transcription factors. Furthermore, tight junction production, arachidonic acid metabolism, leukocyte transendothelial migration, renin angiotensin system, and ECM receptor interaction were upregulated and regulation of autophagy was downregulated in cluster 2. These results suggest that patients in cluster 2 had a violent development of CVD (Figures 7A, B).

### Immune Cell Infiltration Analysis in ICM

ICM is characterized by diffuse fibrosis of the myocardium resulting from prolonged myocardial ischemia due to atherosclerosis of the coronary artery. To clean damaged or dead cells from the infarct site, an inflammatory cascade of signals is triggered, leading to the infiltration of immune cells. However, chronic activation of the immune system can also lead to adverse myocardial effects. Therefore, modulating the infiltration of immune cells to regulate the immune response seems to be a practical way to control disease deterioration.

CIBERSORTx is a deconvolution algorithm that can calculate the infiltration proportion of 22 types of immune cells according to the LM22 reference matrix. First, an independent expression matrix (GSE_merge) was formed after four datasets of ICM were combined, and batch effects were removed. Furthermore, the PCA diagram showed that batch effects from different datasets of ICM were removed (Supplementary Figures 1A, B). Eventually, the independent expression matrix was uploaded into CIBERSORTx to calculate the proportion of immune cells. Compared with the normal controls, the violin plot showed that the proportion of resting T cells CD4 memory (*P* = 0.001), resting NK cells (*P* = 0.002), macrophages M0 (*P* = 0.013), dendritic cells resting (*P* = 0.027), and mast cell activation (*P* = 0.008) increased. However, the proportion of T cells CD4 memory activated (*P* = 0.046) decreased in the ICM samples (Supplementary Figure 2).

**FIGURE 8:**
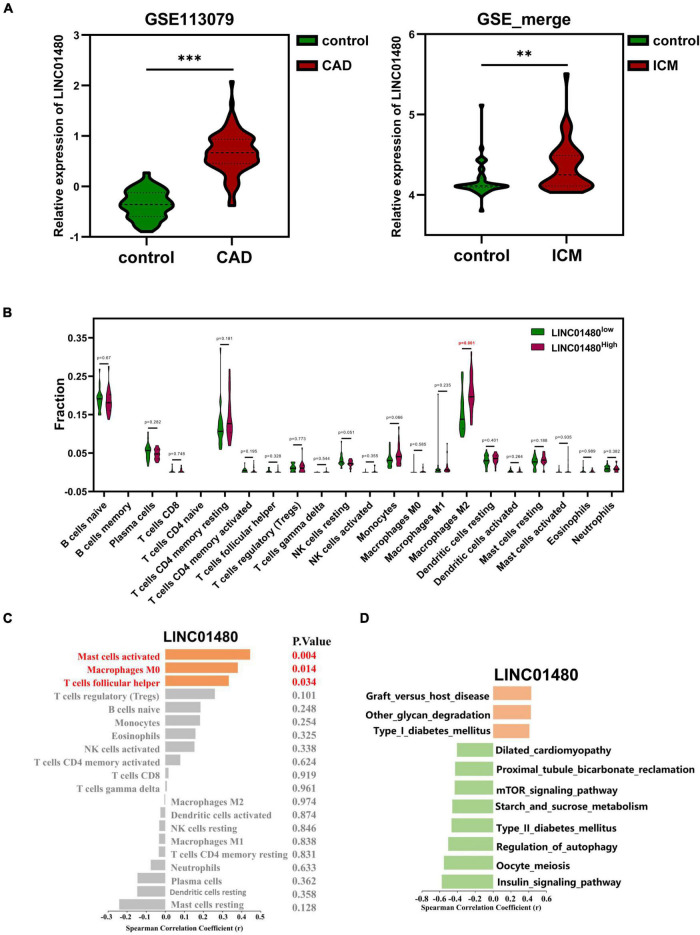
Correlation of the proportion of infiltrating immune cell with the expression of LINC01480. **(A)** LINC01480 showed significant upregulation both in CAD dataset (GSE113079) and ICM datasets (GSE_merge, matrix of the merged and removing batch effects of GSE48166, GSE116250, GSE46224, and GSE120825). **(B)** Violin plot showing the ratio of differentiation of 22 kinds of immune cells among the ICM samples with low or high expression levels of LINC01480 relative to the median of LINC01480 expression level; Wilcoxon rank-sum test was used as the significance test. The red mark indicates the difference in the infiltration between the two groups of samples. **(C)** Spearman’s correlation analysis of LINC01480 and the infiltrating immune cells; immune cells with statistically significant were marked in red color with *P* < 0.05. **(D)** Spearman’s correlation analysis of LINC01480 and GSVA scores of the KEGG pathways; the statistically significant pathways were visualized with *P* < 0.01. CAD, coronary artery disease; ICM, ischemic cardiomyopathy; KEGG, Kyoto Encyclopedia of Genes and Genomes; GSVA, gene set variable analysis. 0.001 < **P < 0.01, ***P < 0.001.

Notably, we found that the expression of LINC01480 was upregulated in ICM left ventricles (Figure 8A). Therefore, we investigated whether LINC01480 affects the infiltration of immune cells. First, we classified the immune cell infiltration matrix of ICM samples into high- and low-expression groups using the median LINC01480 expression value. The results suggested that the infiltration proportion of M2 macrophages (*P* = 0.001) was significantly increased in the LINC01480-highly expressed group (Figure 8B). Subsequently, we found that LINC01480 was positively correlated with mast cell activation (cor = 0.444, *P* = 0.004), macrophages M0 (cor = 0.380, *P* = 0.014), and follicular helper T cells (cor = 0.333, *P* = 0.034) in the ICM samples by calculating the Spearman’s correlation (Figure 8C).

Then, we primarily speculated the biological pathways involving LINC01480 in ICM. By calculating the Spearman’s correlation between the expression value of LINC01480 and the GSVA score of pathways, we found that LINC01480 was positively correlated with the GSVA scores of graft versus host disease (cor = 0.429, *P* = 0.005), other types of glycan degradation (cor = 0.424, *P* = 0.006), and type I diabetes mellitus (cor = 0.407, *P* = 0.008), and the GSVA score of dilated cardiomyopathy (cor = –0.406, *P* = 0.008), proximal tubule bicarbonate reclamation (cor = –0.427, *P* = 0.005), mTOR signaling pathway (cor = –0.431, *P* = 0.005), starch and sucrose metabolism (cor = –0.459, *P* = 0.003), type II diabetes mellitus (cor = –0.468, *P* = 0.002), regulation of autophagy (cor = –0.505, *P* = 0.001), and insulin signaling pathway (cor = –0.578, *P* = 0.001) were negatively correlated with the expression of LINC01480 (Figure 8D).

Finally, we also predicted the function of LINC01480 by analyzing its co-expression genes. We totally identified 2193 positive co-expression genes and 1133 negative co-expression genes by Pearson’s analysis. The results indicated that the KEGG pathways of immune cell adhension, migration, and activation were positively correlated with LINC01480 by analyzing the positive co-expression genes (Supplementary Table 6). On the other hand, multiple heart diseases, the insulin signaling pathway, and Type II diabetes mellitus were negatively correlated with LINC01480 by analyzing the negative co-expression genes. This result is similar to previous results from GSVA analysis, which demonstrated that LINC01480 might promote immune cell infiltration and inhibit the insulin signaling pathway to play a protective role in ICM (Supplementary Table 7).

In conclusion, the immune cell infiltration analysis and pathway analysis suggested that LINC01480 might play a protective role in the progression of ICM.

## Discussion

CAD accounts for the largest proportion of CVDs and causes serious damage to the body, affecting the patient’s quality of life. ICM is considered a special type or advanced stage of CAD and is the major cause of heart failure (HF). Atherosclerosis is the underlying pathological process of CAD, which involves a series of immune cells and immune molecules. Accumulating evidence indicates that many aberrantly expressed lncRNAs participate in multiple atherosclerosis processes, including disturbances in lipid metabolism (36, 37), endothelial cell dysregulation (38), and foam cell formation (39). The low cost of next-generation sequencing technologies leads to more sequencing data being stored in public databases, which allows researchers to easily explore gene interactions and crucial molecules. This study aimed to systematically analyze immune-related lncRNAs in CAD and investigate the relationship between hub immune-related lncRNAs and immune cell infiltration in ICM, which might provide new insights for targeted therapy and drug design.

We first identified DELs and DEIRGs based on the GSE113079 dataset and immune gene list from the ImmPort database. As expected, both the upregulated and downregulated immune mRNAs were enriched in a series of immune-related pathways, such as cytokine production, leukocyte differentiation, NK cell mediated cytotoxicity, and T cell receptor signaling pathway. This indicates that disordered immune-related genes and pathways are involved in the development of CAD, which is consistent with previous reports. Subsequently, a weighted co-expression network was constructed to investigate the expression patterns of DELs and DEIRGs. The constructed immune-related lncRNA-mRNA weighted co-expression network comprised 377 lncRNAs and 119 mRNAs. To our knowledge, this is the first time that immune-related lncRNAs have been systematically studied through co-expression network analysis in CAD. RF-RFE is also a popular feature selection method that searches for the optimal feature set by removing the feature with the lowest influence on the performance of the random forest model from the feature set during each iteration. LASSO logistic regression is a classical method for feature selection, the principle of which is to eliminate some variables using the penalty argument-lambda. Therefore, we used the RF-RFE and LASSO logistic regression algorithms to further screen crucial lncRNAs. Eventually, we identified AL359237.1 and LINC01480 as hub lncRNAs by combining the two algorithms, which were all upregulated in CAD samples.

In the original article on GSE113079 by Li et al., the authors identified 1210 lncRNAs and 890 differentially expressed mRNAs from the expression profile and validated seven lncRNAs. Subsequently, they identified two novel lncRNAs (ENST00000444488.1 and uc010yfd.1) as biomarkers for CAD diagnosis (24). In addition, new lncRNAs were uncovered by mining the GSE113079 again. For example, Zhang et al. found that 12 circulating upregulated and 1 downregulated apoptosis- and autophagy-related lncRNAs could be diagnostic biomarkers for CAD (40). Sun et al. found that lnc-KCNC3-3:1, an upregulated lncRNA, could alleviate the development of atherosclerosis *via* downregulation of the JAK1/STAT3 signaling pathway (41). Xu et al. found that eight epithelial-mesenchymal transition-related lncRNAs are potential biomarkers in CAD (42). Liu et al. found that AC010082.1 and AC011443.1 could be biomarkers of CAD based on the ceRNA mechanism and logistic stepwise regression prediction model, and they validated that these lncRNAs were upregulated in CADs using RT-PCR (43). In this study, we also uncovered two new lncRNAs (AL359237.1 and LINC01480) by mining the GSE113079 dataset, which might predict the progression of atherosclerosis. Unlike others, our focus is on the lncRNAs that are involved in immune regulation in CADs, and these two lncRNAs are closely related to the immune system.

Furthermore, we found that CAD samples could be divided into two clusters by k-means, and PCA and Hclust cluster analysis suggested that it was reasonable. Due to the lack of clinical information, the direct correlation between hub lncRNAs and disease progression could not be explored. It might be a good idea to infer the risk of disease by pathway enrichment analysis for different subgroups. Further enrichment analysis showed that cardiovascular disease-related pathways, such as dilated cardiomyopathy, arrhythmogenic right ventricular cardiomyopathy, and hypertrophic cardiomyopathy, were positively correlated with both subgroups, which suggested that the pathway enrichment analysis method was reliable. In addition, calcium signaling pathway and cell adhesion molecules were also activated, and the T cell receptor signaling pathway was inhibited in both subgroups. Previous studies have demonstrated that these pathways play a critical role in atherosclerosis. For example, dysregulation of the calcium signaling pathway might result in an imbalance of intracellular calcium homeostasis, inducing turbulence in cardiomyocyte contraction and leading to the development of different CVDs (44). The adhesion molecules that are highly expressed on the surface of endothelial cells recruit immune cells to injured sites, which is the initial event in atherosclerosis development (45).

It is worth noting that patients in cluster 2 were involved in more cardiovascular risk pathways. For example, arachidonic acid metabolism, leukocyte transendothelial migration, renin angiotensin system (RAS), and ECM receptor interaction are activated, but the regulation of autophagy is inhibited. The main function of RAS is to maintain the homeostasis of the body environment, and the RAS can promote cardiac and vascular damage by triggering inflammation and structural remodeling under pathophysiological conditions (46). If the degradation of the extracellular matrix is faster than its synthesis, it will make the plaque more prone to rupture because of its thinner fiber cap (47). Furthermore, the composition of the cardiac extracellular matrix due to mechanical stress contributes to the pathogenesis of HF (48). Therefore, we speculate that cluster 2 had advanced disease progression and was prone to exhibit symptoms. Notably, we found that LINC01480 was upregulated in ICM tissues. Therefore, CIBERSORTx was used to further investigate the relationship between LINC01480 and immune cell infiltration. The results showed that the proportion of M2 macrophages was increased in the LINC01480-highly expressed samples. M2 is considered a class of anti-inflammatory macrophages, suggesting that LINC01480 plays an anti-inflammatory role with an increase in its expression level.

By analyzing the correlation between LINC01480 and immune cells, it was found that LINC01480 was significantly positively correlated with the activated mast cells, macrophages (M0), and T follicular helper cells. In addition, the KEGG pathway analysis by GSVA scores and co-expression genes suggested that LINC01480 was positively correlated with type I diabetes mellitus and some pathways involving in immune cell adhesion, migration and activation, and it was negatively correlated with mutiple heart diseases, mTOR signaling pathway, type II diabetes mellitus and the insulin signaling pathway. Type II diabetes mellitus is a classical risk factor for atherosclerosis and is closely correlated with high cholesterol levels and high blood pressure. The Framingham Heart Study suggested that type II diabetes mellitus could increase the risk of CVD by 2- to 3-folds (49). Furthermore, mTOR is a key mediator of the insulin signaling pathway. Therefore, short-term treatment with rapamycin, an mTOR inhibitor, is a promising treatment strategy for acute myocardial infarction and cardiac hypertrophy (50). Based on the results of previous studies, we speculated that LINC01480 inhibits the development of ICM by downregulating the mTOR signaling pathway to reduce the risk of type II diabetes mellitus. We also hypothesized that it would promote the infiltration of M0 and induce its further differentiation into M2, thereby acting as an anti-inflammatory agent.

Previous studies indicated that LINC01480 is dysregulated in multiple cancers (51–55). For example, Chen et al. found that LINC01480 was specifically upregulated in endometrial cancer and could distinguish endometrial cancer from ovarian and cervical cancers (52). Furthermore, Yuan et al. revealed that LINC01480 is an immune microenvironment-related lncRNA in lung adenocarcinoma based on the ESTIMATE method to evaluate the degree of immune infiltration and correlation analysis (54). This is consistent with our finding that LINC01480 plays an important role in immune cell infiltration. However, LINC01480 has not been reported in CVD. Therefore, our findings may provide a direction to investigate it in CAD.

In summary, we systematically analyzed the immune-related lncRNAs in CAD *via* WGCNA. Moreover, we identified two novel lncRNAs (LINC01480 and AL359237.1) that are closely related to the heterogeneity in CAD. Furthermore, we found that LINC01480 was upregulated in the ICM samples, which might play a protective role in ICM. Therefore, our results have laid the foundation for further exploration of the mechanism underlying the immune system dysfunction as well as the roles of immune-related lncRNAs in CAD. Finally, it should be emphasized that there are lacking of relative wet experiments to validate our findings. A large number of clinical patient fellow-up data are need to confirm the feasibility of clustering, and more cellular experiments are necessary to perform for further exploring the functional mechanism of these two lncRNAs in CAD. Only in this way, can LINC01480 be developed as a truly valuable marker for clinical diagnosis and treatment in the future.

## Data Availability Statement

The datasets presented in this study can be found in online repositories. The names of the repository/repositories and accession number(s) can be found in the article/Supplementary Material.

## Author Contributions

QL, TX, and YL were responsible for the overall design and investigation. TX performed data analysis. TX, BX, YW, and QL were responsible for manuscript writing and participated in discussion of the results. All authors contributed to the article and approved the submitted version.

## Conflict of Interest

The authors declare that the research was conducted in the absence of any commercial or financial relationships that could be construed as a potential conflict of interest.

## Publisher’s Note

All claims expressed in this article are solely those of the authors and do not necessarily represent those of their affiliated organizations, or those of the publisher, the editors and the reviewers. Any product that may be evaluated in this article, or claim that may be made by its manufacturer, is not guaranteed or endorsed by the publisher.

## Footnotes

1
https://www.ncbi.nlm.nih.gov/geo/

2
https://www.immport.org/shared/home

3
https://metascape.org/

4
https://www.gsea-msigdb.org/gsea/downloads.jsp

## References

[1] Wong ND. Epidemiological studies of CHD and the evolution of preventive cardiology. *Nat Rev Cardiol.* (2014). 11:276–89. 10.1038/nrcardio.2014.26 24663092

[2] Álvarez-Álvarez MM Zanetti D Carreras-Torres R Moral P Athanasiadis G. A survey of sub-Saharan gene flow into the Mediterranean at risk loci for coronary artery disease. *Eur J Hum Genet.* (2017) 25:472–476. 10.1038/ejhg.2016.200 28098150 PMC5386420

[3] Boudoulas KD Triposciadis F Geleris P Boudoulas H. Coronary atherosclerosis: pathophysiologic basis for diagnosis and management. *Prog Cardiovasc Dis.* (2016) 58:676–92. 10.1016/j.pcad.2016.04.003 27091673

[4] Pantely G Bristow JD. Ischemic cardiomyopathy. *Prog Cardiovasc Dis.* (1984) 27:95–114. 10.1016/0033-0620(84)90021-5 6147879

[5] Sekulic M Zacharias M Medalion B. Ischemic cardiomyopathy and heart failure. *Cir Heart Fail.* (2019) 12:e006006. 10.1161/CIRCHEARTFAILURE.119.006006 31113224

[6] Rizzacasa B Amati F Romeo F Novelli G Mehta JL. Epigenetic modification in coronary atherosclerosis: JACC review topic of the week. *J Am Coll Cardiol.* (2019) 74:1352–65. 10.1016/j.jacc.2019.07.043 31488273

[7] Gistera A Hansson GK. The immunology of atherosclerosis. *Nat Rev Nephrol.* (2017) 13:368–80. 10.1038/nrneph.2017.51 28392564

[8] Bennett MR Sinha S Owens GK. Vascular smooth muscle cells in atherosclerosis. *Circ Res.* (2016) 118:692–702. 10.1161/CIRCRESAHA.115.306361 26892967 PMC4762053

[9] Soehnlein O Libby P. Targeting inflammation in atherosclerosis - from experimental insights to the clinic. *Nat Rev Drug Discov.* (2021) 11:1–22. 10.1038/s41573-021-00198-1 33976384 PMC8112476

[10] Bar C Chatterjee S Thum T. Long noncoding RNAs in cardiovascular pathology, diagnosis, and therapy. *Circulation.* (2016) 134:1484–99. 10.1161/CIRCULATIONAHA.116.023686 27821419

[11] Beermann J Piccoli MT Viereck J Thum T. Non-coding RNAs in development and disease: background, mechanisms, and therapeutic approaches. *Physiol Rev.* (2016) 96:1297–325. 10.1152/physrev.00041.2015 27535639

[12] Dey BK Mueller AC Dutta A. Long non-coding RNAs as emerging regulators of differentiation, development, and disease. *Transcription.* (2014) 5:e944014. 10.4161/21541272.2014.944014 25483404 PMC4581346

[13] Guo F Wu Q Li P Zheng L Ye S Dai X The role of the LncRNA-FA2H-2-MLKL pathway in atherosclerosis by regulation of autophagy flux and inflammation through mTOR-dependent signaling. *Cell Death Differ.* (2019) 26:1670–87. 10.1038/s41418-018-0235-z 30683918 PMC6748100

[14] Wang Y Song X Li Z Liu B. Long non-coding RNAs in coronary atherosclerosis. *Life Sci.* (2018) 211:189–97. 10.1016/j.lfs.2018.08.072 30195033

[15] Zhuang J Peng W Li H Wang W Wei Y Li W Methylation of p15INK4b and expression of ANRIL on chromosome 9p21 are associated with coronary artery disease. *PLoS One.* (2012) 7:e47193. 10.1371/journal.pone.0047193 23091611 PMC3473029

[16] Cho H Shen GQ Wang X Wang F Archacki S Li Y Long noncoding RNA ANRIL regulates endothelial cell activities associated with coronary artery disease by up-regulating CLIP1, EZR, and LYVE1 genes. *J Biol Chem.* (2019) 294:3881–98. 10.1074/jbc.RA118.005050 30655286 PMC6422082

[17] Zhang C Ge S Gong W Xu J Guo Z Liu Z LncRNA ANRIL acts as a modular scaffold of WDR5 and HDAC3 complexes and promotes alteration of the vascular smooth muscle cell phenotype. *Cell Death Dis.* (2020) 11:435. 10.1038/s41419-020-2645-3 32513988 PMC7280314

[18] Zhu Y Yang T Duan J Mu N Zhang T. MALAT1/miR-15b-5p/MAPK1 mediates endothelial progenitor cells autophagy and affects coronary atherosclerotic heart disease *via* mTOR signaling pathway. *Aging (Albany NY).* (2019) 11:1089–109. 10.18632/aging.101766 30787203 PMC6402525

[19] Wang L Qi Y Wang Y Tang H Li Z Wang Y LncRNA MALAT1 suppression protects endothelium against oxLDL-Induced inflammation *via* inhibiting expression of MiR-181b target gene TOX. *Oxid Med Cell Longev.* (2019) 2019:8245810. 10.1155/2019/8245810 31949884 PMC6942911

[20] Toraih EA El-Wazir A Alghamdi SA Alhazmi AS El-Wazir M Abdel-Daim MM Association of long non-coding RNA MIAT and MALAT1 expression profiles in peripheral blood of coronary artery disease patients with previous cardiac events. *Genet Mol Biol.* (2019) 42:509–18. 10.1590/1678-4685-GMB-2018-0185 31188931 PMC6905438

[21] Ye ZM Yang S Xia YP Hu RT Chen SC Li BW LncRNA MIAT sponges miR-149-5p to inhibit efferocytosis in advanced atherosclerosis through CD47 up-regulated. *Cell Death Dis.* (2019) 10:138. 10.1038/s41419-019-1409-4 30755588 PMC6372637

[22] Sun G Li Y Ji Z. Up-regulation of MIAT aggravates the atherosclerotic damage in atherosclerosis mice through the activation of PI3K/Akt signaling pathway. *Drug Deliv.* (2019) 26:641–9. 10.1080/10717544.2019.1628116 31237148 PMC6598488

[23] Barrett T Wilhite SE Ledoux P Evangelista C Kim IF Tomashevsky M NCBI GEO: archive for functional genomics data sets-update. *Nucleic Acids Res.* (2013) 41:D991–5. 10.1093/nar/gks1193 23193258 PMC3531084

[24] Li L Wang L Li H Han X Chen S Yang B Characterization of LncRNA expression profile and identification of novel LncRNA biomarkers to diagnose coronary artery disease. *Atherosclerosis.* (2018) 275:359–67. 10.1016/j.atherosclerosis.2018.06.866 30015300

[25] Yang KC Yamada KA Patel AY Topkara VK George I Cheema FH Deep RNA sequencing reveals dynamic regulation of myocardial noncoding RNAs in failing human heart and remodeling with mechanical circulatory support. *Circulation.* (2014) 129:1009–21. 10.1161/CIRCULATIONAHA.113.003863 24429688 PMC3967509

[26] Sweet ME Cocciolo A Slavov D Jones KL Sweet JR Graw SL Transcriptome analysis of human heart failure reveals dysregulated cell adhesion in dilated cardiomyopathy and activated immune pathways in ischemic heart failure. *BMC Genomics.* (2018) 19:812. 10.1186/s12864-018-5213-9 30419824 PMC6233272

[27] Miao Q Hill MC Chen F Mo Q Ku AT Ramos C SOX11 and SOX4 drive the reactivation of an embryonic gene program during murine wound repair. *Nat Commun.* (2019) 10:4042. 10.1038/s41467-019-11880-9 31492871 PMC6731344

[28] Bhattacharya S Dunn P Thomas CG Smith B Schaefer H Chen J ImmPort, toward repurposing of open access immunological assay data for translational and clinical research. *Sci Data.* (2018) 5:180015. 10.1038/sdata.2018.15 29485622 PMC5827693

[29] Ritchie ME Phipson B Wu D Hu Y Law CW Shi W Limma powers differential expression analyses for RNA-sequencing and microarray studies. *Nucleic Acids Res.* (2015) 43:e47. 10.1093/nar/gkv007 25605792 PMC4402510

[30] Langfelder P Horvath S. WGCNA: an R package for weighted correlation network analysis. *BMC Bioinform.* (2008) 9:559. 10.1186/1471-2105-9-559 19114008 PMC2631488

[31] Shannon P Markiel A Ozier O Baliga NS Wang JT Ramage D Cytoscape: a software environment for integrated models of biomolecular interaction networks. *Genome Res.* (2003) 13:2498–504. 10.1101/gr.1239303 14597658 PMC403769

[32] Yan ZB Yao Y. Variable selection method for fault isolation using least absolute shrinkage and selection operator (LASSO). *Chemometr Intell Lab.* (2015) 146:136–46. 10.1016/j.chemolab.2015.05.019

[33] Darst BF Malecki KC Engelman CD. Using recursive feature elimination in random forest to account for correlated variables in high dimensional data. *BMC Genet.* (2018) 19:65. 10.1186/s12863-018-0633-8 30255764 PMC6157185

[34] Subramanian A Tamayo P Mootha VK Mukherjee S Ebert BL Gillette MA Gene set enrichment analysis: a knowledge-based approach for interpreting genome-wide expression profiles. *Proc Natl Acad Sci U S A.* (2005) 102:15545–50. 10.1073/pnas.0506580102 16199517 PMC1239896

[35] Newman AM Steen CB Liu CL Gentles AJ Chaudhuri AA Scherer F Determining cell type abundance and expression from bulk tissues with digital cytometry. *Nat Biotechnol.* (2019) 37:773–82. 10.1038/s41587-019-0114-2 31061481 PMC6610714

[36] Halley P Kadakkuzha BM Faghihi MA Magistri M Zeier Z Khorkova O Regulation of the apolipoprotein gene cluster by a long noncoding RNA. *Cell Rep.* (2014) 6:222–30. 10.1016/j.celrep.2013.12.015 24388749 PMC3924898

[37] Hu YW Zhao JY Li SF Huang JL Qiu YR Ma X RP5-833A20.1/miR-382-5p/NFIA-dependent signal transduction pathway contributes to the regulation of cholesterol homeostasis and inflammatory reaction. *Arterioscler Thromb Vasc Biol.* (2015) 35:87–101. 10.1161/ATVBAHA.114.304296 25265644

[38] Wu ZH He YY Li DL Fang X Shang T Zhang HK Long noncoding RNA MEG3 suppressed endothelial cell proliferation and migration through regulating miR-21. *Am J Transl Res.* (2017) 9:3326–35. 28804550 PMC5553882

[39] Li Y Sun T Shen S Wang L Yan J. LncRNA DYNLRB2-2 inhibits THP-1 macrophage foam cell formation by enhancing autophagy. *Biol Chem.* (2019) 400:1047–57. 10.1515/hsz-2018-0461 30903747

[40] Zhang L Lou D He D Wang Y Wu Y Cao X Dysregulated circulating apoptosis- and autophagy-related lncRNAs as diagnostic markers in coronary artery disease. *Biomed Res Int.* (2021) 2021:5517786. 10.1155/2021/5517786 34513991 PMC8426068

[41] Sun L He X Zhang T Tao G Wang X. Knockdown of lnc-KCNC3-3:1 alleviates the development of atherosclerosis *via* downregulation of JAK1/STAT3 signaling pathway. *Front Cardiovasc Med.* (2021) 8:701058. 10.3389/fcvm.2021.701058 34540913 PMC8446538

[42] Xiang X Zou RC Liu XY Su QQ. Epithelial-mesenchymal transition-related lncRNAs and SNAI2 are potential biomarkers in coronary artery disease. *Preprint.* (2021): 10.21203/rs.3.rs-940366/v2

[43] Liu C Liu L Gao J Wang J Liu Y. Identification of two long non-coding RNAs AC010082.1 and AC011443.1 as biomarkers of coronary heart disease based on logistic stepwise regression prediction model. *Front Genet.* (2021) 12:780431. 10.3389/fgene.2021.780431 34868268 PMC8637336

[44] Wang R Wang M He S Sun G Sun X. Targeting calcium homeostasis in myocardial ischemia/reperfusion injury: an overview of regulatory mechanisms and therapeutic reagents. *Front Pharmacol.* (2020) 11:872. 10.3389/fphar.2020.00872 32581817 PMC7296066

[45] Galkina E Ley K. Vascular adhesion molecules in atherosclerosis. *Arterioscler Thromb Vasc Biol.* (2007) 27:2292–301. 10.1161/ATVBAHA.107.149179 17673705

[46] Paz Ocaranza M Riquelme JA Garcia L Jalil JE Chiong M Santos RAS Counter-regulatory renin-angiotensin system in cardiovascular disease. *Nat Rev Cardiol.* (2020) 17:116–29. 10.1038/s41569-019-0244-8 31427727 PMC7097090

[47] Solanki A Bhatt LK Johnston TP. Evolving targets for the treatment of atherosclerosis. *Pharmacol Ther.* (2018) 187:1–12. 10.1016/j.pharmthera.2018.02.002 29414673

[48] Zhang YY Bauersachs J Langer HF. Immune mechanisms in heart failure. *Eur J Heart Fail.* (2017) 19:1379–89. 10.1002/ejhf.942 28891154

[49] Fox CS Coady S Sorlie PD D’Agostino RB Pencina MJ Vasan RS Increasing cardiovascular disease burden due to diabetes mellitus - The Framingham Heart Study. *Circulation.* (2007) 115:1544–50. 10.1161/CIRCULATIONAHA.106.658948 17353438

[50] Suhara T Baba Y Shimada BK Higa JK Matsui T. The mTOR signaling pathway in myocardial dysfunction in Type 2 diabetes mellitus. *Curr Diabetes Rep.* (2017) 17:38. 10.1007/s11892-017-0865-4 28434143 PMC8219468

[51] Cui L Chen S Wang D Yang Q. LINC01116 promotes proliferation and migration of endometrial stromal cells by targeting FOXP1 *via* sponging miR-9-5p in endometriosis. *J Cell Mol Med.* (2021) 25:2000–12. 10.1111/jcmm.16039 33372387 PMC7882988

[52] Chen BJ Byrne FL Takenaka K Modesitt SC Olzomer EM Mills JD Transcriptome landscape of long intergenic non-coding RNAs in endometrial cancer. *Gynecol Oncol.* (2017) 147:654–62. 10.1016/j.ygyno.2017.10.006 29050779

[53] Zhang Y Jin T Shen H Yan J Guan M Jin X. Identification of Long Non-Coding RNA expression profiles and co-expression genes in thyroid carcinoma based on The Cancer Genome Atlas (TCGA) database. *Med Sci Monit.* (2019) 25:9752–69. 10.12659/MSM.917845 31856144 PMC6931392

[54] Yuan L Li F Wang S Yi H Li F Mao Y. Identification of tumor microenvironment-related prognostic lncRNAs in lung adenocarcinoma. *Front Oncol.* (2021) 11:719812. 10.3389/fonc.2021.719812 34408984 PMC8366027

[55] Chen S Liang H Hu G Yang H Zhou K Xu L Differently expressed long noncoding RNAs and mRNAs in TK6 cells exposed to low dose hydroquinone. *Oncotarget.* (2017) 8:95554–67. 10.18632/oncotarget.21481 29221148 PMC5707042

