# Supplementary material for: Upregulation of the Long Non-coding RNA LINC01480 Is Associated With Immune Infiltration in Coronary Artery Disease Based on an Immune-Related lncRNA-mRNA Co-expression Network
Source: Front Cardiovasc Med. 2022 Apr 26;9:724262. doi: 10.3389/fcvm.2022.724262 (PMC9086407; doi:10.3389/fcvm.2022.724262)
Supplement: Supplementary file 1 [file Table_1.DOCX]

| **Supplementary Table 1:**GEO datasets used in this study | | | |
| --- | --- | --- | --- |
| Dataset | Type | Sample size | Source |
| GSE113079 | mircoarray | 93CAD 48control | PBMCs |
| GSE48166 | RNAseq | 15ICM 15control | left ventricle |
| GSE116250 | RNAseq | 13ICM 14control | left ventricle |
| GSE46224 | RNAseq | 8ICM 8control | left ventricle |
| GSE120825 | RNAseq | 5ICM 5ICM | left ventricle |

**Supplementary Table 2:** The top 20 upregulated and downregulated lncRNAs in CADs compared with healthy controls

| lncRNA | logFC | AveExpr | t | P-value | adj.P.Val | B |
| --- | --- | --- | --- | --- | --- | --- |
| AC022140.1 | 2.487381629 | -1.880695769 | 10.00106194 | 3.33E-18 | 4.81E-17 | 30.67895597 |
| LINC02731 | 2.015389533 | 3.370414526 | 14.53542133 | 5.05E-30 | 4.28E-28 | 57.67394005 |
| AC024257.1 | 1.869916681 | -3.044429325 | 9.584476275 | 3.95E-17 | 4.96E-16 | 28.23095855 |
| CLMAT3 | 1.844496934 | -2.071824285 | 11.76552355 | 8.24E-23 | 2.12E-21 | 41.19333095 |
| LINC01391 | 1.812533809 | -3.275946504 | 16.67862306 | 1.95E-35 | 6.56E-33 | 70.03856292 |
| AC024619.3 | 1.79244945 | -0.409076817 | 13.52228206 | 2.10E-27 | 1.05E-25 | 51.68892974 |
| AC010082.1 | 1.767075353 | -0.392553395 | 17.95929619 | 1.44E-38 | 1.64E-35 | 77.18770588 |
| AC013248.1 | 1.701847869 | -1.674923686 | 10.88648732 | 1.65E-20 | 3.09E-19 | 35.93563998 |
| AC012358.4 | 1.685693573 | -1.64319686 | 10.28910732 | 5.97E-19 | 9.25E-18 | 32.38233595 |
| TPRG1-AS1 | 1.656397264 | -1.95295181 | 13.65737379 | 9.37E-28 | 5.17E-26 | 52.4909361 |
| LINC00922 | 1.654511603 | -1.924934551 | 11.94126666 | 2.85E-23 | 7.90E-22 | 42.24604812 |
| CYMP-AS1 | 1.619378225 | -1.333874868 | 16.51183743 | 5.06E-35 | 1.45E-32 | 69.09345448 |
| LINC01091 | 1.615724616 | -3.142866123 | 13.89890987 | 2.21E-28 | 1.38E-26 | 53.92207613 |
| AL929601.1 | 1.614412313 | -0.412856434 | 13.34557845 | 6.06E-27 | 2.90E-25 | 50.63832648 |
| AC113167.1 | 1.598645551 | -3.660701297 | 16.72214404 | 1.52E-35 | 5.80E-33 | 70.28466285 |
| LMO7DN | 1.560323834 | -2.133592617 | 8.600153531 | 1.24E-14 | 1.16E-13 | 22.54741684 |
| AC008759.2 | 1.558223076 | -2.232698506 | 13.69145289 | 7.64E-28 | 4.32E-26 | 52.6930832 |
| AC007406.3 | 1.526788544 | -0.526384463 | 12.77724114 | 1.84E-25 | 6.79E-24 | 47.24910428 |
| AL359237.1 | 1.510849596 | -2.872897502 | 16.13678443 | 4.36E-34 | 1.06E-31 | 66.95692223 |
| LINC01432 | 1.476941843 | -2.673919489 | 14.66222337 | 2.39E-30 | 2.16E-28 | 58.41760183 |
| AP005328.1 | -2.572517955 | 0.261104545 | -9.674799693 | 2.31E-17 | 3.00E-16 | 28.75996708 |
| AC079790.2 | -2.17971761 | -1.400324955 | -12.85528982 | 1.15E-25 | 4.40E-24 | 47.71532277 |
| AL391244.1 | -1.790620765 | -0.536556187 | -6.87716397 | 1.73E-10 | 1.01E-09 | 13.14280821 |
| AC068790.6 | -1.72434518 | -2.02185489 | -12.92910758 | 7.39E-26 | 2.91E-24 | 48.15606406 |
| AC012640.4 | -1.704478844 | -3.657994821 | -13.11848865 | 2.37E-26 | 1.01E-24 | 49.28579423 |
| HIPK1-AS1 | -1.684096161 | -2.046354167 | -9.381365312 | 1.31E-16 | 1.54E-15 | 27.04537377 |
| MED14OS | -1.662596019 | -2.04796746 | -9.799774471 | 1.10E-17 | 1.49E-16 | 29.49359434 |
| AL359220.1 | -1.63441222 | 6.103376035 | -9.736257972 | 1.61E-17 | 2.12E-16 | 29.12050405 |
| AC012557.2 | -1.634038511 | -1.564154766 | -6.886357959 | 1.65E-10 | 9.60E-10 | 13.19048177 |
| AC008870.3 | -1.628178539 | -3.598821427 | -8.38983015 | 4.13E-14 | 3.66E-13 | 21.3566254 |
| AL078645.1 | -1.619499709 | -3.899211654 | -11.8044479 | 6.52E-23 | 1.71E-21 | 41.42648417 |
| PACERR | -1.610081192 | -2.705706861 | -5.942652468 | 2.02E-08 | 9.30E-08 | 8.479357274 |
| AL355490.2 | -1.607330996 | 0.006562861 | -7.452528634 | 7.83E-12 | 5.30E-11 | 16.18451072 |
| AC117500.2 | -1.603367698 | -2.909366599 | -9.577138318 | 4.12E-17 | 5.16E-16 | 28.18802782 |
| AZIN1-AS1 | -1.583691199 | -0.488741533 | -4.515712658 | 1.30E-05 | 4.27E-05 | 2.211017885 |
| AC026770.1 | -1.542479586 | -1.225989296 | -5.674901253 | 7.38E-08 | 3.18E-07 | 7.217221468 |
| LINC02810 | -1.53437415 | -4.345513312 | -9.14020136 | 5.38E-16 | 5.84E-15 | 25.64556744 |
| AL137779.2 | -1.53131538 | -0.818884526 | -6.864501444 | 1.85E-10 | 1.07E-09 | 13.07720171 |
| AC244502.1 | -1.528706786 | 0.833294091 | -8.892594496 | 2.28E-15 | 2.31E-14 | 24.21835759 |
| AC002456.1 | -1.524364169 | -3.680091241 | -8.283335341 | 7.57E-14 | 6.51E-13 | 20.75747347 |

**Supplementary Table 3:** The top 20 upregulated and downregulated mRNAs in CADs compared with healthy controls

| mRNA | logFC | AveExpr | t | P-value | adj.P.Val | B |
| --- | --- | --- | --- | --- | --- | --- |
| CFAP74 | 2.203130607 | -0.118208731 | 11.28517356 | 1.56E-21 | 4.30E-20 | 38.29876228 |
| ACTBL2 | 2.080142373 | 1.544968009 | 12.4265235 | 1.60E-24 | 7.25E-23 | 45.11796081 |
| BIRC7 | 1.819073751 | -2.532215589 | 16.9137639 | 5.65E-36 | 3.84E-33 | 71.2613883 |
| KIF17 | 1.77919636 | -1.800474971 | 18.62459307 | 4.13E-40 | 1.24E-36 | 80.68842129 |
| SAXO1 | 1.754529638 | -2.123614482 | 11.96635952 | 2.56E-23 | 9.30E-22 | 42.36871539 |
| NMNAT2 | 1.750952974 | -2.478076523 | 14.44163373 | 9.44E-30 | 1.05E-27 | 57.05954377 |
| NEURL1B | 1.722449745 | -2.748976231 | 16.40678612 | 1.01E-34 | 4.34E-32 | 68.40098847 |
| HOXA3 | 1.712806032 | 3.206484757 | 15.71533928 | 5.44E-33 | 1.34E-30 | 64.45414519 |
| FTMT | 1.696639783 | -0.913484479 | 14.87017405 | 7.55E-31 | 1.07E-28 | 59.56396623 |
| SHANK1 | 1.687355323 | 1.114250678 | 18.73888532 | 2.21E-40 | 8.29E-37 | 81.30527337 |
| ARPIN | 1.684985526 | 0.130496716 | 8.032985611 | 3.17E-13 | 2.30E-12 | 19.37435155 |
| OPN4 | 1.669340379 | -1.412649684 | 17.80234364 | 3.84E-38 | 4.80E-35 | 76.20230112 |
| TRPM5 | 1.655911042 | -2.247921778 | 15.80727824 | 3.19E-33 | 8.35E-31 | 64.98185932 |
| NUPR1 | 1.65470941 | -2.882781251 | 15.60373028 | 1.04E-32 | 2.36E-30 | 63.81236572 |
| OR4C3 | 1.545271075 | -0.753370853 | 16.29288995 | 1.95E-34 | 6.94E-32 | 67.75440681 |
| AVPR1B | 1.544817338 | -2.526246876 | 13.64375 | 1.08E-27 | 8.05E-26 | 52.35878043 |
| PYDC2 | 1.536425562 | -3.033564167 | 12.88029568 | 1.05E-25 | 5.86E-24 | 47.82445136 |
| SLC19A3 | 1.523404297 | 2.090292544 | 17.54588792 | 1.61E-37 | 1.72E-34 | 74.78601438 |
| NOG | 1.50262714 | -0.045358685 | 13.5474412 | 1.92E-27 | 1.37E-25 | 51.78848416 |
| CPEB1 | 1.492622277 | -3.28471726 | 14.68602179 | 2.23E-30 | 2.78E-28 | 58.48967553 |
| ARHGEF33 | -2.827416924 | -3.538938689 | -10.58855581 | 1.02E-19 | 2.15E-18 | 34.15003537 |
| PAK2 | -2.337363292 | -2.558890116 | -8.577463482 | 1.43E-14 | 1.27E-13 | 22.42851421 |
| RBPJL | -2.30564452 | 2.530342993 | -8.727620771 | 6.04E-15 | 5.69E-14 | 23.28234829 |
| IL1A | -2.180862306 | -3.225535405 | -5.443310617 | 2.21E-07 | 7.27E-07 | 6.18551606 |
| C22orf31 | -2.161530218 | -1.355204972 | -11.30729542 | 1.36E-21 | 3.82E-20 | 38.43081899 |
| FAM47A | -1.911942236 | -3.56879758 | -10.83476952 | 2.34E-20 | 5.42E-19 | 35.61374699 |
| FAM169A | -1.89887833 | -1.704815409 | -15.14966701 | 1.47E-31 | 2.52E-29 | 61.1886584 |
| KRT33B | -1.870928759 | -3.518158868 | -8.59110232 | 1.33E-14 | 1.18E-13 | 22.50587728 |
| BNC2 | -1.866152908 | -1.766515145 | -12.20316646 | 6.15E-24 | 2.47E-22 | 43.78387165 |
| NELFA | -1.851593782 | -3.089084179 | -8.641439843 | 9.92E-15 | 9.03E-14 | 22.7917392 |
| SH2D4A | -1.784182484 | -1.124516653 | -7.488939023 | 6.49E-12 | 3.93E-11 | 16.39941739 |
| PDGFD | -1.78021374 | -1.828739045 | -10.62014497 | 8.47E-20 | 1.80E-18 | 34.3376275 |
| CD300A | -1.725958842 | -0.497212301 | -8.436822398 | 3.21E-14 | 2.69E-13 | 21.63307735 |
| B3GNT7 | -1.718705343 | -2.846490004 | -10.04207635 | 2.68E-18 | 4.41E-17 | 30.91663967 |
| AKAP5 | -1.684726653 | -0.073495402 | -12.30438131 | 3.34E-24 | 1.42E-22 | 44.38852836 |
| DLEU7 | -1.670714721 | -2.690023523 | -8.260345839 | 8.76E-14 | 6.92E-13 | 20.64120851 |
| CCL20 | -1.66392095 | -2.847614674 | -4.218483979 | 4.34E-05 | 0.000105548 | 1.096609475 |
| SMURF1 | -1.65246114 | -3.097830742 | -9.943756965 | 4.81E-18 | 7.58E-17 | 30.33773207 |
| XKR7 | -1.629609398 | -1.656372105 | -9.974759949 | 4.00E-18 | 6.38E-17 | 30.52017184 |
| CKLF | -1.606631782 | -3.661721235 | -8.981045615 | 1.39E-15 | 1.46E-14 | 24.73331802 |

**Supplementary Table 4:** Comparison of several hub gene screening methods

| **RF-RFE (10-fold CV)** | **RF-RFE(Bootstrap)** | **LASSO (10-fold CV)** |
| --- | --- | --- |
| EFCAB6-AS1 | AC022007.1 | LINC02747 |
| LINC01820 | EFCAB6-AS1 | AC113167.1 |
| AC022007.1 | LINC01820 | ZNF426-DT |
| TSPAN9-IT1 | LINC01391 | LINC01391 |
| **AL359237.1*** | LINC02747 | AC084116.3 |
| AL136146.2 | AL031847.1 | **AL359237.1*** |
| AP003059.2 | **AL359237.1*** | **LINC01480*** |
| AL03847.1 | TSPAN9-IT1 | AC010319.3 |
| SLC2A1-AS1 | AP003059.2 | LINC02731 |
| **LINC01480*** | NTM-IT | AC005906.2 |
| AL136146.2 | **LINC01480*** | AC147651.2 |
|  | AL136146.2 | AZIN1-AS1 |
|  | SLC2A1-AS1 |  |
|  | ZNF426-DT |  |
|  | LINC02143 |  |
|  | AC113167.1 |  |

Note. The crucial variables were screened by RF-RFE (10-fold cross validation), RF-RFE (Bootstrap) and LASSO (10-fold cross validation), respectively. RF-RFE: The random forest-recursive feature elimination; LASSO: The least absolute shrinkage and selection operator logistic regression. Two LASSO (LINC01480 and AL359237.1) were the overlapped molecules appearing together in three algorithms and marked with star.

**Supplementary Table 5:** Classification results of hierarchical clustering and K-means

| **sample** | **K-means** | **Hclust** | **sample** | **K-means** | **Hclust** | **sample** | **K-means** | **Hclust** |
| --- | --- | --- | --- | --- | --- | --- | --- | --- |
| GSM3095926 | 1 | 1 | GSM3095964 | 1 | 1 | GSM3095957 | 2 | 2 |
| GSM3095927 | 1 | 1 | GSM3095965 | 1 | 1 | GSM3095969 | 2 | 2 |
| GSM3095928 | 1 | 1 | GSM3095966 | 1 | 1 | GSM3095971 | 2 | 1 |
| GSM3095929 | 1 | 1 | GSM3095967 | 1 | 1 | GSM3095974 | 2 | 1 |
| GSM3095930 | 1 | 1 | GSM3095968 | 1 | 1 | GSM3095975 | 2 | 2 |
| GSM3095931 | 1 | 1 | GSM3095970 | 1 | 1 | GSM3095983 | 2 | 2 |
| GSM3095932 | 1 | 1 | GSM3095972 | 1 | 1 | GSM3095985 | 2 | 2 |
| GSM3095933 | 1 | 1 | GSM3095973 | 1 | 1 | GSM3095986 | 2 | 2 |
| GSM3095934 | 1 | 1 | GSM3095976 | 1 | 1 | GSM3095987 | 2 | 2 |
| GSM3095937 | 1 | 1 | GSM3095977 | 1 | 1 | GSM3095988 | 2 | 2 |
| GSM3095939 | 1 | 1 | GSM3095978 | 1 | 1 | GSM3095989 | 2 | 2 |
| GSM3095940 | 1 | 1 | GSM3095979 | 1 | 1 | GSM3095991 | 2 | 2 |
| GSM3095941 | 1 | 1 | GSM3095980 | 1 | 1 | GSM3095996 | 2 | 2 |
| GSM3095942 | 1 | 1 | GSM3095981 | 1 | 1 | GSM3095997 | 2 | 2 |
| GSM3095944 | 1 | 1 | GSM3095982 | 1 | 1 | GSM3096000 | 2 | 2 |
| GSM3095945 | 1 | 1 | GSM3095984 | 1 | 1 | GSM3096001 | 2 | 2 |
| GSM3095946 | 1 | 1 | GSM3095990 | 1 | 1 | GSM3096003 | 2 | 2 |
| GSM3095947 | 1 | 1 | GSM3095992 | 1 | 1 | GSM3096004 | 2 | 2 |
| GSM3095948 | 1 | 1 | GSM3095993 | 1 | 1 | GSM3096005 | 2 | 2 |
| GSM3095949 | 1 | 1 | GSM3095994 | 1 | 1 | GSM3096006 | 2 | 2 |
| GSM3095951 | 1 | 1 | GSM3095995 | 1 | 1 | GSM3096007 | 2 | 2 |
| GSM3095952 | 1 | 1 | GSM3095998 | 1 | 1 | GSM3096008 | 2 | 2 |
| GSM3095953 | 1 | 1 | GSM3095999 | 1 | 1 | GSM3096009 | 2 | 2 |
| GSM3095954 | 1 | 1 | GSM3096002 | 1 | 1 | GSM3096010 | 2 | 2 |
| GSM3095955 | 1 | 1 | GSM3096011 | 1 | 1 | GSM3096012 | 2 | 2 |
| GSM3095958 | 1 | 1 | GSM3095935 | 2 | 1 | GSM3096013 | 2 | 2 |
| GSM3095959 | 1 | 1 | GSM3095936 | 2 | 2 | GSM3096014 | 2 | 2 |
| GSM3095960 | 1 | 1 | GSM3095938 | 2 | 2 | GSM3096015 | 2 | 2 |
| GSM3095961 | 1 | 1 | GSM3095943 | 2 | 2 | GSM3096016 | 2 | 2 |
| GSM3095962 | 1 | 1 | GSM3095950 | 2 | 2 | GSM3096017 | 2 | 2 |
| GSM3095963 | 1 | 1 | GSM3095956 | 2 | 2 | GSM3096018 | 2 | 2 |

**Supplementary Table 6:** The enriched pathways that were positively correlated with LINC01480 by Kyoto Encyclopedia of Genes and Genomes pathway analysis.

| **Terms** | **Counts** | **P-value** |
| --- | --- | --- |
| **hsa04514: Cell adhesion molecules (CAMs)** | 47 | 1.41E-12 |
| hsa05150: Staphylococcus aureus infection | 24 | 1.50E-09 |
| **hsa04660: T cell receptor signaling pathway** | 30 | 3.17E-07 |
| hsa04064:NF-kappa B signaling pathway | 25 | 8.60E-06 |
| hsa04062: Chemokine signaling pathway | 41 | 1.03E-05 |
| hsa04611: Platelet activation | 30 | 8.50E-05 |
| **hsa04512: ECM-receptor interaction** | 23 | 8.68E-05 |
| hsa04650: Natural killer cell mediated cytotoxicity | 28 | 1.70E-04 |
| hsa04610: Complement and coagulation cascades | 19 | 2.47E-04 |
| hsa04640: Hematopoietic cell lineage | 21 | 6.94E-04 |
| hsa04672: Intestinal immune network for IgA production | 14 | 9.73E-04 |
| hsa04510: Focal adhesion | 38 | 0.001022086 |
| hsa04060: Cytokine-cytokine receptor interaction | 43 | 0.001073933 |
| hsa05323: Rheumatoid arthritis | 20 | 0.002061634 |
| hsa05340: Primary immunodeficiency | 11 | 0.00223821 |
| **hsa04940: Type I diabetes mellitus** | 12 | 0.003763127 |
| hsa04020: Calcium signaling pathway | 32 | 0.004501026 |
| hsa04974: Protein digestion and absorption | 19 | 0.0049314 |
| hsa04662: B cell receptor signaling pathway | 16 | 0.00548964 |
| hsa04142: Lysosome | 23 | 0.008755754 |
| hsa04015: Rap1 signaling pathway | 35 | 0.008787561 |
| **hsa04670: Leukocyte trans endothelial migration** | 22 | 0.009796158 |

**Supplementary Table 7:** The enriched pathways that were negatively correlated with LINC01480 by Kyoto Encyclopedia of Genes and Genomes pathway analysis.

| **Term** | **Count** | **P-value** |
| --- | --- | --- |
| hsa00190: Oxidative phosphorylation | 51 | 1.61E-25 |
| hsa04260: Cardiac muscle contraction | 32 | 1.30E-17 |
| hsa01200: Carbon metabolism | 33 | 1.11E-12 |
| hsa01100: Metabolic pathways | 141 | 8.27E-12 |
| hsa00020: Citrate cycle (TCA cycle) | 15 | 1.83E-09 |
| hsa00620: Pyruvate metabolism | 14 | 1.16E-06 |
| hsa00071: Fatty acid degradation | 13 | 1.35E-05 |
| **hsa05414: Dilated cardiomyopathy** | 18 | 3.55E-05 |
| **hsa05410: Hypertrophic cardiomyopathy (HCM)** | 17 | 5.06E-05 |
| hsa01212: Fatty acid metabolism | 13 | 5.85E-05 |
| hsa03050: Proteasome | 12 | 1.19E-04 |
| hsa04120: Ubiquitin mediated proteolysis | 22 | 3.14E-04 |
| hsa04261: Adrenergic signaling in cardiomyocytes | 22 | 3.48E-04 |
| **hsa04910: Insulin signaling pathway** | 22 | 3.48E-04 |
| **hsa05412: Arrhythmogenic right ventricular cardiomyopathy (ARVC)** | 14 | 4.53E-04 |
| hsa00010: Glycolysis / Gluconeogenesis | 14 | 4.53E-04 |
| hsa04146: Peroxisome | 15 | 0.001200864 |
| hsa01230: Biosynthesis of amino acids | 13 | 0.002929369 |
| hsa00280: Valine, leucine and isoleucine degradation | 10 | 0.003691353 |
| **hsa04931: Insulin resistance** | 15 | 0.013529825 |
| hsa03320: PPAR signaling pathway | 11 | 0.013785561 |
| **hsa04930: Type II diabetes mellitus** | 9 | 0.014106663 |
| hsa04922: Glucagon signaling pathway | 13 | 0.034049123 |
| hsa04152: AMPK signaling pathway | 15 | 0.037316405 |
