# Supplementary material for: Upregulation of the Long Non-coding RNA LINC01480 Is Associated With Immune Infiltration in Coronary Artery Disease Based on an Immune-Related lncRNA-mRNA Co-expression Network
Source: Front Cardiovasc Med. 2022 Apr 26;9:724262. doi: 10.3389/fcvm.2022.724262 (PMC9086407; doi:10.3389/fcvm.2022.724262)
Supplement: Supplementary file 2 [file Data_Sheet_1.DOCX]

**Abbreviations**

| CAD | Coronary artery disease |
| --- | --- |
| lncRNA | Long noncoding RNA |
| ICM | Ischaemic cardiomyopathy |
| CVDs | Cardiovascular diseases |
| LDL | Low-density lipoprotein |
| OxLDL | Oxidized LDL |
| ANRIL | Antisense noncoding RNA in the INK4 locus |
| MALAT1 | Metastasis-associated lung adenocarcinoma transcript 1 |
| MIAT | Myocardial infarction associated transcript |
| mTOR | Mammalian target of rapamycin |
| PBMCs | Peripheral blood mononuclear cells |
| GEO | Gene Expression Omnibus |
| IRGs | Immune-related genes |
| ENA | European Nucleotide Archive |
| VST | Variance stabilizing transformed |
| PCA | Principal component analysis |
| DEMs | Differentially expressed immune mRNAs |
| DELncs | Differentially expressed lncRNAs |
| DEIRGs | Differentially expressed immune-related genes |
| WGCNA | Weighted gene co-expression network analysis |
| LASSO | The least absolute shrinkage and selection operator |
| RF-RFE | Random forest-recursive feature elimination |
| GSEA | Gene set enrichment analysis |
| GSVA | Gene set variation analysis |
| KEGG | The Kyoto Encyclopedia of Genes and Genomes |
| HF | Heart failure |
