# Supplementary material for: Upregulation of the Long Non-coding RNA LINC01480 Is Associated With Immune Infiltration in Coronary Artery Disease Based on an Immune-Related lncRNA-mRNA Co-expression Network
Source: Front Cardiovasc Med. 2022 Apr 26;9:724262. doi: 10.3389/fcvm.2022.724262 (PMC9086407; doi:10.3389/fcvm.2022.724262)
Supplement: Supplementary file 3 [file Data_Sheet_2.DOCX]

**
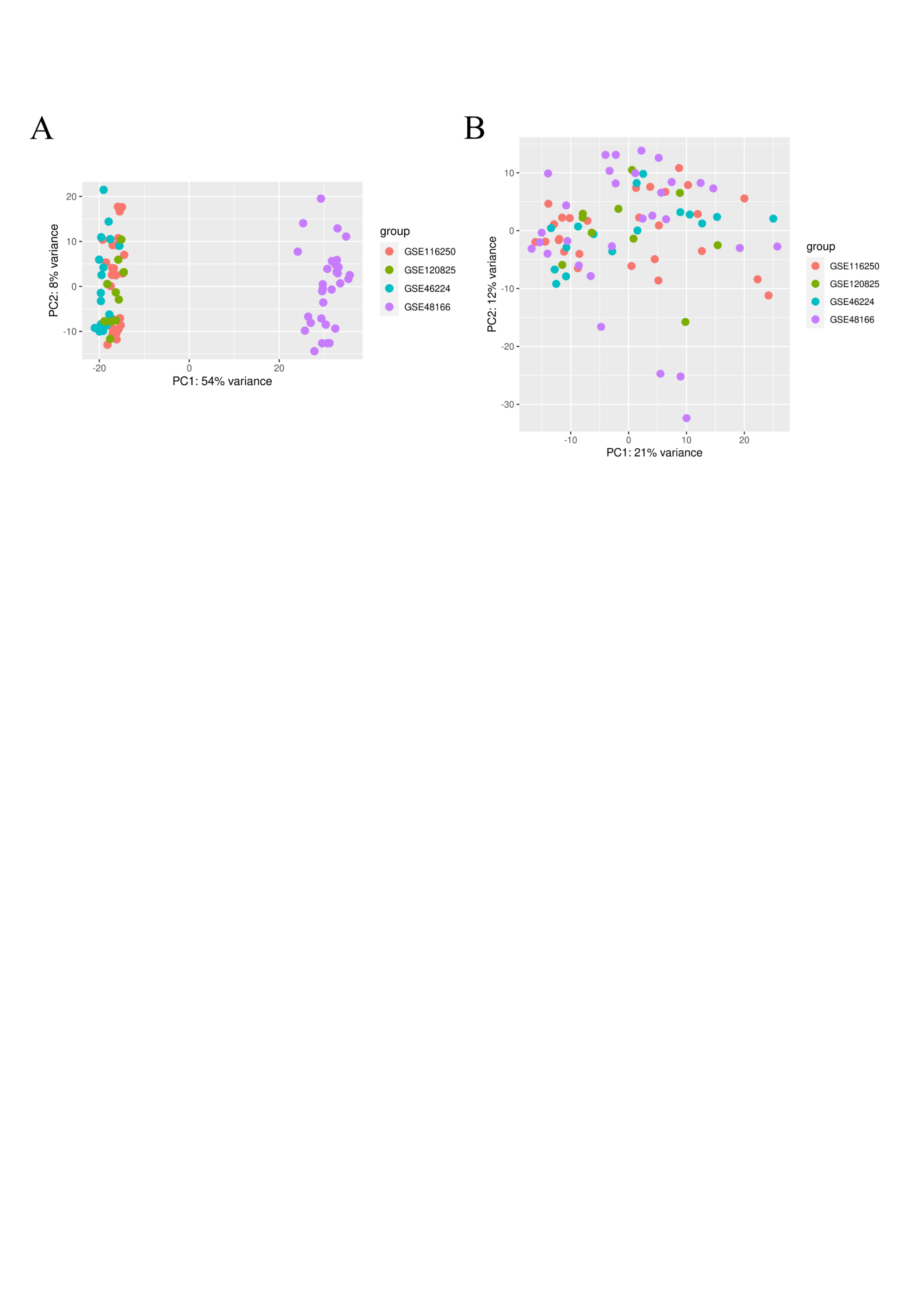
**

**Supplementary Figure 1:** Two-dimensional PCA cluster plot of sample correction. The PCA cluster plot before (**A**) and after (**B**) sample correction. Different colors represent the samples derived from corresponding datasets. PCA: principal component analysis.
