# Supplementary material for: Upregulation of the Long Non-coding RNA LINC01480 Is Associated With Immune Infiltration in Coronary Artery Disease Based on an Immune-Related lncRNA-mRNA Co-expression Network
Source: Front Cardiovasc Med. 2022 Apr 26;9:724262. doi: 10.3389/fcvm.2022.724262 (PMC9086407; doi:10.3389/fcvm.2022.724262)
Supplement: Supplementary file 4 [file Data_Sheet_3.DOCX]

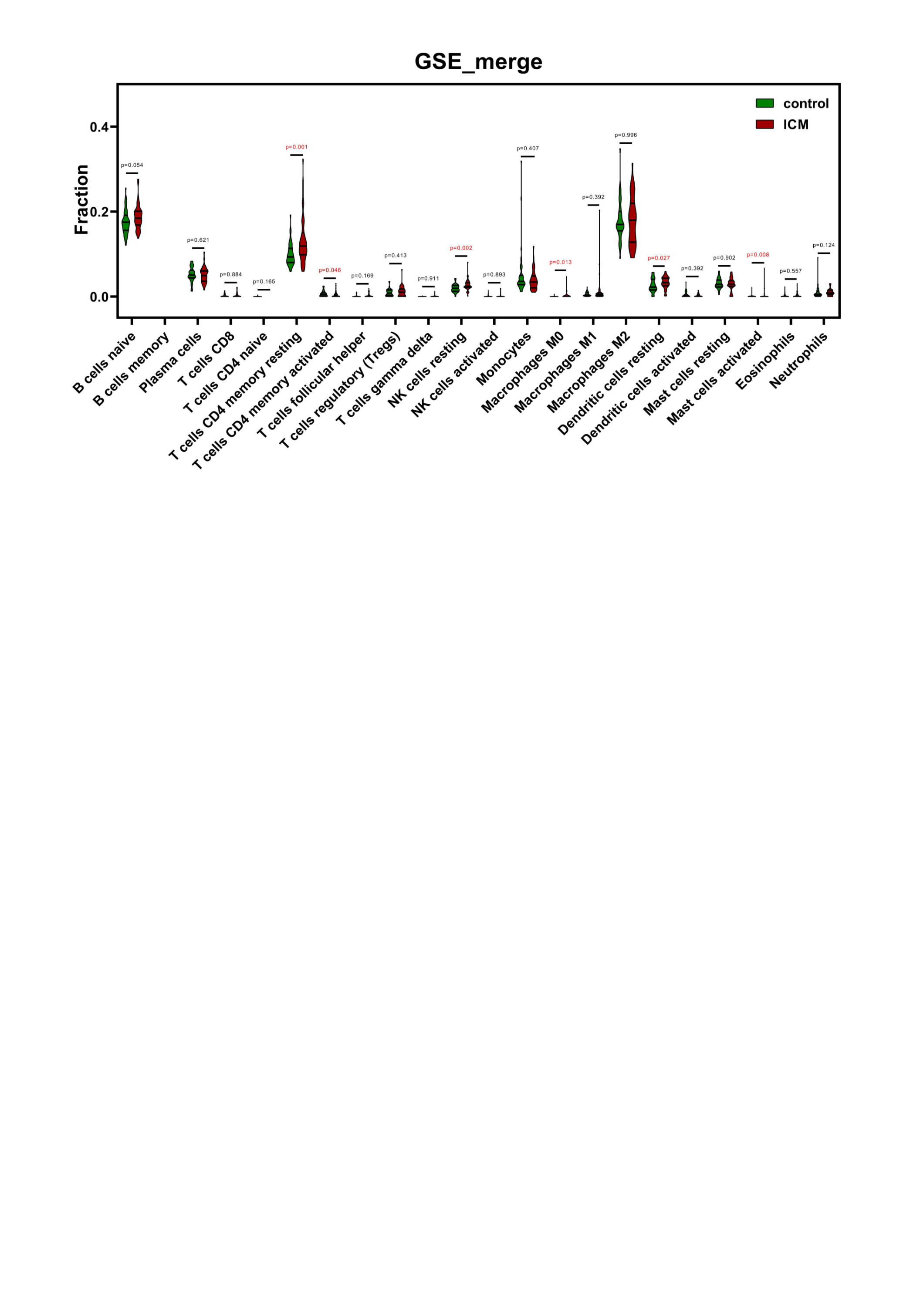
**Supplementary Figure 2:** Immune cell infiltration analysis in the ICM samples. Violin plot shows the ratio differentiation of 22 kinds of immune cells between the ICM samples and controls; Wilcoxon rank-sum test was used as the significance test. The red mark indicates the difference in the infiltration between the two groups of samples. ICM: ischemic cardiomyopathy.
